# Comorbidities, Endocrine Medications, and Mortality in Prader–Willi Syndrome—A Swedish Register Study

**DOI:** 10.3390/jcm14041307

**Published:** 2025-02-16

**Authors:** Julia Giesecke, Anna Oskarsson, Maria Petersson, Anna Skarin Nordenvall, Giorgio Tettamanti, Ann Nordgren, Charlotte Höybye

**Affiliations:** 1 Department of Molecular Medicine and Surgery, Karolinska Institute, 171 77 Stockholm, Sweden; julia.giesecke@ki.se (J.G.); anna.skarin-nordenvall@ki.se (A.S.N.); giorgio.tettamanti@ki.se (G.T.); ann.nordgren@ki.se (A.N.); charlotte.hoybye@ki.se (C.H.); 2 Department of Endocrinology, Karolinska University Hospital, 171 76 Stockholm, Sweden; 3 Department of Endocrinology, St Göran’s Hospital, 112 19 Stockholm, Sweden; 4 Department of Radiology, Karolinska University Hospital, 171 76 Stockholm, Sweden; 5 Unit of Epidemiology, Institute of Environmental Medicine, Karolinska Institute, 171 77 Stockholm, Sweden; 6 Department of Clinical Genetics and Genomics, Karolinska University Hospital, 171 76 Stockholm, Sweden; 7 Department of Laboratory Medicine, Institute of Biomedicine, University of Gothenburg, 405 30 Göteborg, Sweden; 8 Department of Clinical Genetics and Genomics, Sahlgrenska University Hospital, 413 45 Göteborg, Sweden

**Keywords:** Prader–Willi syndrome, comorbidity, mortality, medications, obesity

## Abstract

**Background:** Prader–Willi Syndrome (PWS) is a rare, genetic, multi-systemic disorder. Its main characteristics are muscular hypotonia, behavioral problems, intellectual disability, endocrine deficiencies, hyperphagia, and a high risk of morbid obesity and related comorbidities. This study aimed to investigate the rate of comorbidity, prescription of endocrine medications, and mortality in individuals with PWS compared to the general population. **Methods:** The association between PWS and outcomes were investigated in a matched cohort study of individuals born in the period of 1930–2018 with data from Swedish national health and welfare registers. Each individual was matched with 50 non-PWS comparisons. The associations between PWS, outcomes and prescribed endocrine medications were estimated through Cox proportional hazard models, presented as Hazard Ratios (HR) with 95% Confidence Intervals (CIs). **Results:** Among 360 individuals (53% men) with PWS, 16% had diabetes mellitus, 6% heart failure, 4% vein thrombosis, 2% atrial fibrillation, 2% coronary heart disease, and 1% pulmonary embolism. Individuals with PWS had an increased rate of heart failure (HR: 23.85; 95% CI: 14.09–40.38), diabetes mellitus (HR: 17.49; 95% CI: 12.87–23.74), vein thrombosis (HR: 10.44; 95% CI: 5.69–19.13), pulmonary embolism (HR: 5.77; 95% CI: 2.27–14.67), atrial fibrillation (HR: 5.19; 95% CI: 2.48–10.86), and coronary heart disease (HR: 3.46; 95% CI: 1.50–7.97) compared to non-PWS individuals. Somatotropin was prescribed in 63%, antidiabetics in 18%, and thyroid hormones in 16% of the PWS individuals (<1%, 2%, and 3%, respectively, in non-PWS individuals). The rate of mortality was fifteen times higher in PWS than in non-PWS, with a mean age at death of 42 years. **Conclusions:** The rates of diabetes mellitus and cardiovascular comorbidities were higher in individuals with PWS. As expected, the prescription of somatotropin was high, but the endocrine prescription pattern also reflected the high prevalence of diabetes mellitus and thyroid illness. Although the mean age at death was older than previously reported, a higher awareness and intensified efforts to avoid obesity, as well as the prevention and early treatment of cardiovascular and endocrine comorbidity, are crucial aims in the care of people with PWS.

## 1. Introduction

Prader–Willi Syndrome (PWS) is rare, with an estimated prevalence of 1:10,000 to 1:30,000 newborns [1,2]. The syndrome is caused by an absence of the expression of imprinted genes in the paternally derived PWS region (11.2-q13) of chromosome 15 by one of several genetic mechanisms (paternal deletion (frequency 65–70%), maternal uniparental disomy 15 (30–35%), and rarely an imprinting defect (1–5%)) [1].

The syndrome is complex and multi-systemic. PWS is a hypothalamic disorder characterized by muscular hypotonia and feeding difficulties in early infancy, followed in early childhood by excessive eating and the gradual development of morbid obesity, unless food intake is controlled and supervised [2,3]. Body composition is abnormal with more body fat than lean body mass [3]. Motor milestones and language development are delayed, and most individuals have some degree of cognitive impairment. Hypogonadism is present in almost all and manifests as genital hypoplasia, incomplete puberty, and usually infertility. Stature is short if somatotropin (growth hormone, GH) is not administered during childhood. Characteristic facial features, strabismus, and scoliosis are often present. The clinical profile is variable, depending on the age at diagnosis, compliance to diet restrictions and physical activity, treatment of hormone deficiencies, and other therapies [2].

Compared to the general population, life expectancy in PWS is decreased, and until a few decades ago, only a small proportion of individuals with PWS reached the age of 40 years. An annual death rate of 3% in PWS compared to 1% for the general population has been reported [4]; however, a decline in mortality rate over time was noted in a study from the United Kingdom [5].

PWS is associated with several comorbidities such as behavioral and psychiatric problems, obesity, diabetes mellitus, and respiratory and cardiovascular diseases [5]. The somatic comorbidity is often caused by complications related to obesity, which is a central problem in PWS [6]. Concerning obesity, five nutritional phases have been described, from feeding difficulties and a failure to thrive in infancy to progressive hyperphagia with a high risk of severe obesity starting already in childhood [7]. Despite intense research, there is still no specific pharmacologic treatment for hyperphagia. Thus, a frequency of obesity from 40% in children up to 98% in adults has been reported [8]. Independent of the degree of obesity, body composition is abnormal in PWS with more body fat than lean body mass and with most of the fat tissue located subcutaneously [3]. Several studies have reported a better metabolic profile than expected in relation to the BMI [3,9] and this has been attributed to a lower amount of visceral fat tissue, high adiponectin and ghrelin levels, and low levels of GH [9,10]. However, increased fasting insulin and insulin resistance have been observed with an increasing age and BMI in children with PWS [9]. Higher insulin levels and a high HOMA index were also observed in obese as compared to non-obese adults with PWS [11,12]. The frequency of type II diabetes has been reported to be up to 25% [6,11,12]. Muscular hypotonia and decreased muscle mass are other cardinal features of PWS, starting in early infancy and continuing throughout childhood, into adulthood, although it improves with age. It affects body posture, the speed of movement, bone mineral density, metabolism, and energy expenditure [2,13]. Together with hyperphagia, the muscular hypotonia increases the risk for obesity.

A Danish register study involving 155 adults with Prader–Willi Syndrome (PWS) revealed a significantly higher prevalence of various behavioral and cardiovascular conditions compared to the general population [14]. Additionally, a separate study from the Netherlands examining 70 individuals with PWS found that 61% had undiagnosed health issues [15]. Among these individuals, all males and 93% of females had hypogonadism, 74% had scoliosis, 18% had hypertension, 19% had hypercholesterolemia, 17% had type II diabetes, and 17% had hypothyroidism [15].

In summary, previous research has indicated both an increased prevalence of cardiovascular and endocrine comorbidities as well as elevated mortality in PWS individuals.

Using data from several national Swedish health registers, this study aimed to further assess the rate of comorbidity and the prescriptions of antidiabetics, thyroid hormones, and somatotropin and to examine the mortality rate in a nationwide cohort of individuals with PWS and matched non-PWS comparisons.

## 2. Materials and Methods

### 2.1. Swedish Registers

Sweden has a large system of registers in which demographic and healthcare data are collected continuously [16]. All permanent residents are given unique personal identity numbers (PINs) that enables linkage between the registers [17].

Statistics Sweden maintains the Total Population Register (TPR), which provides comprehensive information on all citizens, including on country of birth, deaths, and emigration [18]. Statistics Sweden additionally holds registers from 1990 and onwards with both data on educational level (the Longitudinal Integration Database for Health Insurance and Labor Market Studies, Swedish acronym: LISA) as well as the Multi Generation Register (MGR), enabling linkage between index individuals and their biological and adoptive parents [17,19,20].

The Swedish National Board of Health and Welfare oversees the National Patient Register (NPR), which has been collecting data on in-patient care at public and private hospitals since the 1960s, with nationwide information on in-patient data available since 1987 and hospital outpatient data since 2001 [21]. This board also manages the Cause of Death Register (CDR), which has recorded mortality data since 1952, and the nationwide Medical Birth Registry (MBR), which, since 1973, has recorded data on all births, including information on reproductive history, pregnancy, delivery, and the neonatal period [22,23,24]. The Prescribed Drug Register (PDR), also held by The Swedish National Board of Health and Welfare, contains records of all prescribed medications dispensed at Swedish pharmacies since July 2005 [25].

Furthermore, the clinical genetics department at the Karolinska University Laboratory holds the regional Laboratory Information Management Systems (LIMS) that include genetic diagnoses and disease-causing variants on patients referred to Karolinska for genetic testing [26].

Clinical and biochemical data are not included in the registers.

### 2.2. Identification of Individuals with Prader–Willi Syndrome

Individuals with a diagnosis of PWS born between 1930 and 2018 were identified through the NPR, MBR, and/or LIMS. In 1997, the Swedish National Board of Health and Welfare implemented a specific ICD-10 code for PWS (Q87.1F); consequently, only individuals with a registered diagnosis of PWS between 1997 and 2018 were included in the present study. Three individuals with a genetically confirmed PWS in the LIMS, who were not registered as PWS in the national registers, were added to the PWS cohort. The diagnosis of PWS was validated in a subset of the exposed cohort through review of medical records. Information about specific genotypes was not included in the registers.

Each individual with PWS was matched with 50 comparisons without a PWS diagnosis, identified through the TPR, by year of birth, sex, and Swedish county of birth; for non-Swedish-born residents, the international region of birth was used for matching. Only non-PWS individuals alive at the date of the index individual’s first PWS diagnosis were eligible for randomization. As the aim of the study was to compare individuals with PWS to the general population, no further parameters for matching were used.

For sensitivity analyses, a restricted cohort was selected, consisting of individuals who had at least two recorded diagnoses of PWS and/or genetically confirmed PWS, resulting in a cohort of 261 individuals born in Sweden between 1961 and 2018. This cohort excluded individuals with any other subsequent diagnoses of chromosomal aberrations following the final recorded PWS diagnosis.

The study was approved by the Swedish ethical authority (Registration no: 2018/1849-32).

### 2.3. Outcome and Covariates

Cardiovascular and metabolic outcomes were identified through the NPR and included diabetes mellitus, heart failure, angina pectoris, acute myocardial infarction, coronary heart disease, aortic valve disease, atrial fibrillation, cerebrovascular disease, aortic dissection or aneurysm, vein thrombosis, and pulmonary embolism (ICD-codes are available in Supplementary Table S1). Diabetes mellitus type I and type II are not possible to distinguish from each other in the NPR before ICD-10. Diabetes was therefore categorized according to age at onset, 0–15 and >15, with higher age at onset likely indicating type II diabetes.

Data on prescribed endocrine medications were collected from the PDR and included anti-diabetics, thyroid therapy, and somatotropin treatment according to the Anatomical Therapeutic Chemical Classification system (ATC) (ATC-codes included are available in Table 1, Table 2 and Table 3).

Data on date of death were collected from the CDR. Data on date of birth and international region or Swedish county of birth were collected from the TPR. Parental level of education was used as a proxy for socioeconomic status and was collected from the LISA and MGR held by Statistics Sweden.

### 2.4. Statistics

The association between PWS and the different outcomes of interest was estimated using Cox proportional hazard models with attained age as the underlying time scale. Results were presented as Hazard Ratios (HRs) with 95% Confidence Intervals (CIs), both crude and adjusted for year of birth, sex, and parental educational level. Each individual was followed from birth to outcome event, emigration, and death or end of study period (31 December 2018), whichever occurred first. Data on prescribed medications and causes of death were available for an extended period; therefore, the study period was extended to 31 December 2019 for analyses involving medications and to 31 December 2020 for analyses related to mortality. Only individuals alive and living in Sweden at the start of the PDR (July 2005) were included in analyses concerning medications. Schoenfeld residuals were used to test for proportional hazards. When possible, models stratified by sex and age were applied if the test indicated non-proportional hazards. Numbers (N) less than five were censored from the descriptive tables and marked as NA in analyses. All analyses were performed with Stata statistical software, version 14.

## 3. Results

### 3.1. Cohort Characteristics

**Figure 1:**
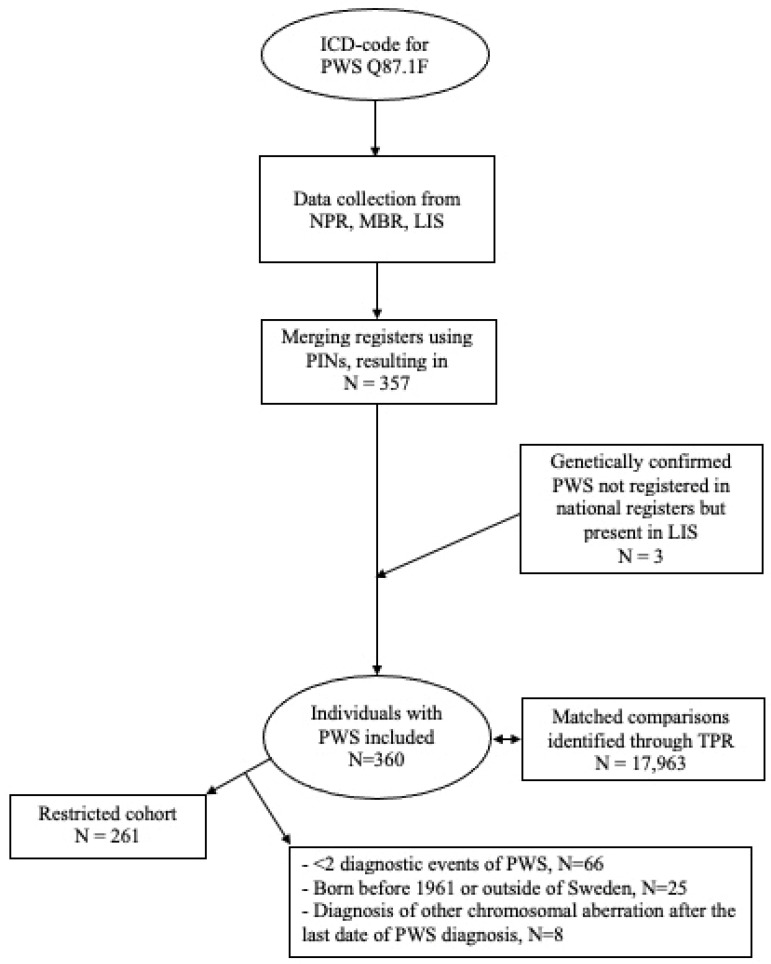
Flowchart showing the definition of the full and the restricted cohort of individuals with PWS included in the study.

The full cohort consisted of 360 individuals with PWS whereof 261 fulfilled the criteria for inclusion in the restricted cohort (Figure 1). In the full cohort, 53% of the individuals were men, and most of the study population had been born after 1971 (86%) (Table 1). Diabetes mellitus was registered in 16% of PWS individuals as compared to 1% in non-PWS individuals, heart failure in 6% compared to <1% in non-PWS individuals, and vein thrombosis and pulmonary embolism in 4% and 1%, respectively, both being <1% in non-PWS individuals. The mean age at death was 41.7 for PWS and 47.5 in non-PWS individuals. Similar results were obtained for the restricted cohort (Table 2). Further baseline characteristics of both cohorts are presented in Table 1 and Table 2.

### 3.2. Comorbidities

There was a significant association between PWS and diabetes mellitus in the full cohort (adjusted HR: 17.49; 95% CI: 12.87–23.74). In age-stratified analyses, the association remained high in PWS individuals both before and after the age of fifteen, although the association was much higher among those older than fifteen at the first diagnosis of diabetes mellitus (Table 3).

The association between PWS and heart failure was strong (adjusted HR: 23.85; 95% CI: 14.09–40.38) (Table 3), and the association remained in both sexes after sex stratification but was somewhat lower in men (HR: 22.50; 95% CI: 11.77–43.02) (Table 4). There was an increased rate of coronary heart disease in PWS individuals (HR: 3.46; 95% CI: 1.50–7.97) and atrial fibrillation/flutter (HR: 5.19; 95% CI: 2.48–10.86) (Table 3). Furthermore, there was an increased rate of both pulmonary embolism (HR: 5.77; 95% CI: 2.27–14.67) and other venous thromboembolisms (HR: 10.44 95% CI 5.69–19.13) in individuals with PWS, where the association with the latter was stronger among women than men (Table 3 and Table 4). The age at first diagnosis for each of the analyzed cardiovascular diseases was significantly lower in PWS individuals than in the matched comparisons (Table 5). Angina pectoris, myocardial infarction, aortic valve disease, aortic dissection or aneurysm, and cerebrovascular disease were registered in less than five individuals with PWS and therefore omitted from analyses. Similar results were present in the restricted cohort, although many analyses were omitted due to low numbers (n < 5) (Table 3).

### 3.3. Prescribed Endocrine Medications

In analyses conducted on the full cohort but restricted to individuals with PWS alive and living in Sweden from 2005, somatotropin was prescribed in 63%, anti-diabetic medications in 18%, and thyroid hormones in 16% of the individuals with PWS (Table 1). In non-PWS individuals, these medications were prescribed in <1%, 2%, and 3%, respectively. Similar results were seen in the restricted cohort, although antidiabetic medication was prescribed in a somewhat lower frequency (16%) while somatotropin was prescribed in a higher frequency (76%) (Table 2). The association between PWS and prescribed medications was particularly high for thyroid hormone prescription before the age of twenty (HR: 51.5; 95% CI: 32.80–80.99) and for anti-diabetics (HR: 13.18; 95% CI: 9.83–17.68) (Table 3).

### 3.4. Mortality

At the end of the follow up, 11% (n = 41) of individuals with PWS in the full cohort had died, as compared to 1% of the non-PWS (Table 1), as reflected by a significantly increased rate of death by any cause in PWS (HR: 15.03; 95% CI: 10.55–21.41) (Table 3). The all-cause mortality rate was similar for women (HR: 14.43; 95% CI: 8.21–25.36) and men (HR: 15.21; 95% CI: 9.64–24.00) (Table 4).

## 4. Discussion

In this nationwide register-based cohort study, we investigated the association between PWS, cardiovascular and metabolic outcomes, and prescribed endocrine medications. In line with previous studies, we found that individuals with PWS were more likely to have diabetes mellitus, heart failure, and venous thromboembolism. Compared to non-PWS individuals, individuals with PWS had a more-than-twenty-times-higher rate of heart failure, a seventeen-times-higher rate of diabetes mellitus, a ten-times-higher rate of vein thrombosis, and a five-times-higher rate of atrial fibrillation and pulmonary embolism. It was also highly likely that individuals with PWS were prescribed somatotropin, antidiabetics, and thyroid hormones. There was a strong association between PWS and the prescription of thyroid hormones and antidiabetics. In this fairly young cohort (86% of the individuals were born after 1970), 11% of the individuals with PWS had died as compared to only 1% of the non-PWS, resulting in a fifteen-times-higher rate of death in PWS.

In the present study, the prevalence of diabetes mellitus was 16% and the rate of diabetes mellitus was seventeen times higher in PWS than in non-PWS individuals. The risk of diabetes was especially high after the age of fifteen, indicating an expected high proportion of type II diabetes among PWS patients. These results were in line with previous observations and underlined the importance of the prevention of obesity and regular control of glucose metabolism already from childhood, particularly in obese individuals with PWS.

Obesity and diabetes mellitus are well-known risk factors for cardiovascular disease. Previous studies had reported that cardiovascular diseases are frequent in PWS and present already at a young age [27,28]. These results were confirmed by our study. As reported in a case series of four adults with PWS and heart failure, cardiovascular disease can go unnoticed for a long time [29]. Individuals with PWS might not have typical symptoms, and for many of them, it can be difficult to express their symptoms and need for care. Edema, dyspnea, and pain may be caused by obesity itself, as well as by kyphoscoliosis, and the high pain tolerance believed to be associated with the syndrome can cause patient delay. Consequently, the diagnosis of cardiovascular disease can be missed and delayed [29]. In addition, hypogonadism is very common in PWS and it is well known that hypogonadism is associated with poorer general health, a risk of cardiovascular disease, reduced bone density, and a worse general condition [2]. Respiratory diseases are also common in PWS, especially respiratory sleep abnormalities, which include both central and obstructive apnea [30,31]. It is well known that untreated obstructive sleep apnea (OSA) may lead to cardiovascular complications including hypertension, cor pulmonale, and stroke [32]. Again, the prevention of obesity is important to minimize the risk of developing OSA and to keep metabolic parameters and blood pressure normal to liberally evaluate for OSA and to treat it when diagnosed.

Somatotropin was the most frequently prescribed medication of those analyzed in this study. This was not surprising as GH treatment is registered for the treatment of children with PWS and our cohort was relatively young. In most countries, the GH treatment of adults with PWS is not a registered treatment and GH deficiency has to be confirmed before GH treatment can be initiated. GH treatment in children with PWS started in the 1990s, and several studies have shown several very good effects, in particular on height and body composition [33]. In adults, GH no longer causes growth but has beneficial effects on body composition and metabolism [34]. Thus, on GH treatment, lean body mass increases while body fat decreases, physical activity improves, and beneficial effects on cardiovascular risk markers, as well as on behavior and the quality of life, have been reported [2,35,36]. Despite the positive effects, long-term effects are not completely known, neither in PWS nor in other GH-deficient patients. A majority of the individuals with PWS in this study were treated with somatotropin and we can speculate upon whether the longevity, which, in comparison with earlier studies was increased, could be associated with this.

According to the literature, up to 17% of individuals with PWS have hypothyroidism, which may be congenital or can develop at any time after birth but is more frequently developed during childhood [37,38]. In line with this, thyroid hormones were prescribed in 16% of our cohort.

Previous studies had reported a significantly high incidence of all-cause mortality in PWS. The mortality rate has been estimated to be 3% per year compared to 1% in the general population [4]. A systematic review of mortality in PWS reported data from 500 cases, where the average age of death was 22.1 years. The two major causes of death were respiratory (35.0%) and cardiovascular diseases (16.8%); sudden death was reported in 4.2% of cases [39]. The mean age at death in our study was 41.7, a finding that may be partially attributed to the fact that only individuals that had survived until the introduction of ICD-10 were included. Other explanations for the difference between our findings and previous studies may be that it was caused by differences in the BMI, the more intensive treatment of diabetes mellitus, a higher number of individuals being treated with somatotropin, or differences in healthcare systems. Some of the previous studies were also older, which could have contributed to the differences.

The strength of our study was the large cohort of individuals with PWS and the comparison with matched non-PWS individuals, together with the possibility of linking data between different registers. However, it should be noted that several additional comorbidities and endocrine medications were registered and prescribed for this cohort during the observed time period but in too low numbers for robust statistical calculation. In addition, our data were retrieved from registers without the availability of important clinical data, such as on cardiovascular examination, non-medical treatment for diabetes, or parental or other caregiver attempts at preventing weight gain, which could also be presumed to be associated with life expectancy and general health. As a result, the use of sex hormones to treat hypogonadism in PWS individuals could not be assessed as the registers do not specify whether prescribed sex hormones are aimed at treating hypogonadism or as contraceptives in female patients. Although somatotropin treatment constitutes a great change in the treatment of individuals with PWS during the last three decades and is believed to have positive effects on general health, the lack of clinical data prevents us from making reliable statistical analyses comparing different age groups with regard to somatotropin treatment and outcomes.

Finally, some comorbidities (such as obesity and respiratory diseases) might have been considered part of the syndrome and therefore may not have received a separate diagnostic code, i.e., they could have been underreported among individuals with PWS.

## 5. Conclusions

In conclusion, the results from our large national register study of individuals with PWS were in line with those of previous studies, confirming that cardiovascular diseases and diabetes mellitus were more common than in non-PWS individuals and cardiovascular diseases occurred at an earlier age. These diseases are presumably related to obesity and start at a young age. The prescription pattern of medications reflected the high prevalence of endocrine disorders and indicated a positive effect on the outcome, and death occurred at an older age than previously reported. Overall, our study indicated a continuous need for awareness and intensified efforts to avoid obesity together with the prevention and early treatment of cardiovascular and endocrine comorbidity.

## Figures and Tables

**Table 1:** Data on the full cohort of individuals with the diagnosis of PWS born in or out of Sweden between 1930 and 2018.

	PWS		Matched Comparisons	
	N = 360	%	N = 17,963	%
Male	190	52.78%	9463	52.68%
Female	170	47.22%	8500	47.32%
**Birth region**				
Sweden	345	95.83%	17250	96.03%
Other	14	3.89%	700	3.90%
Missing	1	0.28%	13	0.07%
**Birth decade**				
1930–1940	1	0.28%	50	0.28%
1941–1950	8	2.22%	400	2.23%
1951–1960	13	3.61%	650	3.62%
1961–1970	27	7.50%	1350	7.52%
1971–1980	45	12.50%	2250	12.53%
1981–1990	63	17.50%	3150	17.54%
1991–2000	72	20.00%	3600	20.04%
2001–2010	75	20.83%	3750	20.88%
2011–2018	56	15.56%	2763	15.38%
**Death**				
Any cause	41	11.38%	191	1.06%
Mean age at death	41.7 (0.6–80.9)		47.5 (0.0–81.8)	
**Diagnosis**				
Diabetes mellitus	57	15.83%	249	1.39%
Heart failure	22	6.11%	70	0.39%
Angina pectoris	<5	0.28–1.39%	77	0.43%
Acute myocardial infarction	<5	0.28–1.39%	71	0.40%
Coronary heart disease	6	1.67%	133	0.74%
Aortic valve stenosis/insufficiency	<5	0.28–1.39%	34	0.19%
Atrial fibrillation	8	2.22%	119	0.66%
Cerebrovascular disease	<5	0.28–1.39%	120	0.67%
Aortic dissection or aneurysm	0	0.00%	26	0.14%
Vein thrombosis	13	3.61%	89	0.5%
Pulmonary embolism	5	1.39%	51	0.28%
**Prescribed medications**				
A10 (anti-diabetic)	63	18.00%	377	2.11%
H01AC (somatotropin and agonists)	220	62.86%	23	0.13%
H03 (thyroid hormones)	57	16.29%	519	2.90%

**Table 2:** Data on the restricted cohort of individuals with at least two registered PWS diagnoses or genetically confirmed PWS. Born in Sweden, 1961–2018.

	PWS		Matched Comparisons	
	N = 261	%	N = 13,050	%
Male	133	50.96%	6650	50.96%
Female	128	49.04%	6400	49.04%
**Birth decade**				
1961–1970	16	6.13%	800	6.13%
1971–1980	32	12.26%	1600	12.26%
1981–1990	52	19.92%	2600	19.92%
1991–2000	51	19.54%	2550	19.54%
2001–2010	61	23.37%	3050	23.37%
2011–2018	49	18.77%	2450	18.77%
**Diagnosis**				
Diabetes mellitus	37	14.18%	112	0.86%
Heart failure	8	3.07%	17	0.13%
Angina pectoris	0	0.00%	11	0.08%
Acute myocardial infarction	<5	0.38–1.92%	13	0.10%
Coronary heart disease	<5	0.38–1.92%	23	0.18%
Aortic valve stenosis/insufficiency	<5	0.38–1.92%	12	0.09%
Atrial fibrillation	<5	0.38–1.92%	26	0.20%
Cerebrovascular disease	<5	0.38–1.92%	39	0.30%
Aortic dissection or aneurysm	0	0.00%	6	0.05%
Vein thrombosis	<5	0.38–1.92%	45	0.34%
Pulmonary embolism	<5	0.38–1.92%	23	0.20%
**Prescribed medications**				
A10 (diabetic)	42	16.22%	173	1.33%
H01AC (somatotropin and agonists)	196	75.68%	18	0.14%
H03 (thyroid hormones)	41	15.83%	295	2.27%

**Table 3:** Associations between Prader–Willi syndrome and comorbidity, prescribed medications, and mortality in the full and the restricted cohorts.

	Full Cohort		Restricted Cohort	
	HR	95% CI	HR	95% CI
**Diagnosis**				
Diabetes mellitus at any age	17.49	12.87–23.74	21.23	14.40–31.30
Diabetes mellitus 0–15 y.a	4.17	1.67–10.42	5.50	2.18–13.88
Diabetes mellitus > 15 y.a	25.91	18.40–36.48	39.85	24.75–64.15
Coronary heart disease	3.46	1.50–7.97	n.a	n.a
Heart failure	23.85	14.09–40.38	35.92	14.07–91.68
Atrial fibrillation	5.19	2.48–10.86	n.a	n.a
Vein thrombosis	10.44	5.69–19.13	n.a	n.a
Pulmonary embolism	5.77	2.27–14.67	n.a	n.a
**Prescribed drugs (ATC)**				
Anti-diabetics (A10)	13.18	9.83–17.68	15.80	10.99–22.72
Thyroid hormones (H03) 0–20 y.a	51.5	32.80–80.99	49.02	29.96–80.22
Thyroid hormones (H03) > 20 y.a	2.14	1.31–3.49	2.21	1.13–4.32
**Death from any cause**	15.03	10.55–21.41	13.15	7.90–21.89

All analyses are inherently adjusted for sex and birth year. Analyses are additionally adjusted for parental educational level. n.a stands for not applicable.

**Table 4:** Adjusted associations between Prader–Willi syndrome and cardiovascular comorbidity and mortality in the full and restricted cohorts.

	Full Cohort—Male		Full Cohort—Female		Restricted Cohort—Male		Restricted Cohort—Female	
	Adjusted *		Adjusted *		Adjusted *		Adjusted *	
	HR	95% CI	HR	95% CI	HR	95% CI	HR	95% CI
**Diagnosis**								
Heart failure	22.50	11.77–43.02	26.97	10.87–66.92	47.20	14.54–153.23	20.93	3.88–112.86
Venous thromboembolism	8.80	3.32–23.33	11.27	5.17–24.56	n.a	n.a	n.a	n.a
**Death from any cause**	15.21	9.64–24.00	14.43	8.21–25.36	10.41	5.25–20.65	20.33	9.09–45.48

* Adjusted for birth year and highest level of parental education. n.a stands for not applicable.

**Table 5:** Age at first registered diagnosis of analyzed cardiovascular diseases in the full cohort, presented as mean and median ages in years, with standard deviations.

	PWS			Matched Comparisons		
	Mean	Median	SD	Mean	Median	SD
**Diagnosis**						
Coronary heart disease	45.0	44.3	12.2	55.2	55.9	10.5
Heart failure	37.7	35.3	18.6	50.4	58.9	22.8
Atrial fibrillation	45.6	41.1	19.8	54.0	56.9	15.2
Vein thrombosis	41.1	35.5	13.8	42.5	43.4	15.6
Pulmonary embolism	35.7	29.6	16.9	44.1	46.3	16.6

## Data Availability

Anonymized personal data were obtained from national Swedish Registry holders after ethical approval and secrecy assessment. According to Swedish laws and regulations, personal sensitive data can only be made available for researchers who fulfil legal requirements for access to personal sensitive data. Contact Ann Nordgren (ann.nordgren@ki.se) for questions about data access.

## Supplementary Materials

The following supporting information can be downloaded at: https://www.mdpi.com/article/10.3390/jcm14041307/s1, Table S1: ICD-codes.
