# Supplementary material for: Comorbidities, Endocrine Medications, and Mortality in Prader–Willi Syndrome—A Swedish Register Study
Source: J Clin Med. 2025 Feb 16;14(4):1307. doi: 10.3390/jcm14041307 (PMC11856969; doi:10.3390/jcm14041307)
Supplement: Supplementary file 1 [file jcm-14-01307-s001.zip › jcm-3452680-supplementary.pdf]

Supplementary Table S1. ICD-codes.

	**ICD-10**	**ICD-9**	**ICD-8**	**ICD-7**
Diabetes mellitus	E10, E11, E13	250	250	260
Heart failure	I50	428	427.0, 427.4	434.1, 782.40
Angina pectoris	I20	411B, 413	413	420.2
Acute myocardial infarction	I21, I22	410	410	420.1
Coronary heart disease	I25	412, 414	412	420.0
Aortic valve stenosis/insufficiency	I35	424B	424.1	421.1
Atrial fibrillation	I48	427D	427.92	433.12, 433.13
Cerebrovascular disease	I63, I65, I66, G45	436, 433, 434, 435	432, 433, 435	332, 334, 333.99
Aortic dissection or aneurysm	I71	441	441	451
Deep vein thrombosis	I80	451	451	463, 464, 466
PE	I26	415B	450	465
